# Tranexamic acid for acute gastrointestinal bleeding (the HALT-IT trial): statistical analysis plan for an international, randomised, double-blind, placebo-controlled trial

**DOI:** 10.1186/s13063-019-3561-7

**Published:** 2019-07-30

**Authors:** Amy Brenner, Adefemi Afolabi, Syed Masroor Ahmad, Monica Arribas, Rizwana Chaudhri, Timothy Coats, Jack Cuzick, Ian Gilmore, Christopher Hawkey, Vipul Jairath, Kiran Javaid, Aasia Kayani, Muttiullah Mutti, Muhammad Arif Nadeem, Haleema Shakur-Still, Simon Stanworth, Andrew Veitch, Ian Roberts, HALT-IT Trial Collaborators

**Affiliations:** 1 Clinical Trials Unit, Department of Population Health, London School of Hygiene and Tropical Medicine, Keppel Street, London, WC1E 7HT UK; 2 Department of Surgery, College of Medicine, University of Ibadan, University College Hospital, Queen Elizabeth Road, Ibadan, 200001 Nigeria; 3 Department of Medicine Unit III, Jinnah Postgraduate Medical Centre, Rafiq Shaheed Road, Karachi, 75510 Pakistan; 4 Rawalpindi Medical University, Holy Family Hospital, Rawalpindi, Pakistan; 5 Department of Cardiovascular Sciences, University of Leicester, Infirmary Square, Leicester, LE1 5WW UK; 6 Centre for Cancer Prevention, Wolfson Institute of Preventive Medicine, Queen Mary University of London, London, EC1M 6BQ UK; 7 University of Liverpool, Liverpool, UK; 8 Faculty of Medicine and Health Sciences, University of Nottingham, Queens Medical Centre, Nottingham, NG7 2UH UK; 9 Division of Gastroenterology, Department of Medicine, University Hospital, Western University, London, Canada; 10 Department of Epidemiology and Biostatistics, Western University, London, ON Canada; 11 Rawalpindi Medical University and London School of Hygiene and Tropical Medicine (RMU-LSHTM) Collaboration, Room No 294, Holy family Hospital, Said Pur Road, Rawalpindi, Pakistan; 12 Department of Medicine, Services Hospital Unit III, Medical Unit III, Jail Road, Lahore, Pakistan; 13 Transfusion Medicine, NHS Blood and Transplant, Oxford, UK; 14 Department of Haematology, Oxford University Hospitals NHS Foundation Trust, Oxford, UK; 15 Radcliffe Department of Medicine, University of Oxford, and Oxford BRC Haematology Theme, Oxford, UK; 16 Department of Gastroenterology, Royal Wolverhampton NHS Trust, Wolverhampton, UK

**Keywords:** Gastrointestinal haemorrhage, Tranexamic acid, Clinical trial, Statistical analysis

## Abstract

**Background:**

Acute gastrointestinal (GI) bleeding is an important cause of mortality worldwide. Bleeding can occur from the upper or lower GI tract, with upper GI bleeding accounting for most cases. The main causes include peptic ulcer/erosive mucosal disease, oesophageal varices and malignancy. The case fatality rate is around 10% for upper GI bleeding and 3% for lower GI bleeding. Rebleeding affects 5–40% of patients and is associated with a four-fold increased risk of death. Tranexamic acid (TXA) decreases bleeding and the need for blood transfusion in surgery and reduces death due to bleeding in patients with trauma and postpartum haemorrhage. It reduces bleeding by inhibiting the breakdown of fibrin clots by plasmin. Due to the methodological weaknesses and small size of the existing trials, the effectiveness and safety of TXA in GI bleeding is uncertain. The Haemorrhage ALleviation with Tranexamic acid – Intestinal system (HALT-IT) trial aims to provide reliable evidence about the effects of TXA in acute upper and lower GI bleeding.

**Methods:**

The HALT-IT trial is an international, randomised, double-blind, placebo-controlled trial of tranexamic acid in 12,000 adults (increased from 8000) with acute upper or lower GI bleeding. Eligible patients are randomly allocated to receive TXA (1-g loading dose followed by 3-g maintenance dose over 24 h) or matching placebo. The main analysis will compare those randomised to TXA with those randomised to placebo on an intention-to-treat basis, presenting the results as effect estimates (relative risks) and confidence intervals. The primary outcome is death due to bleeding within 5 days of randomisation and secondary outcomes are: rebleeding; all-cause and cause-specific mortality; thromboembolic events; complications; endoscopic, radiological and surgical interventions; blood transfusion requirements; disability (defined by a measure of patient’s self-care capacity); and number of days spent in intensive care or high-dependency units. Subgroup analyses for the primary outcome will consider time to treatment, location of bleeding, cause of bleed and clinical Rockall score.

**Discussion:**

We present the statistical analysis of the HALT-IT trial. This plan was published before the treatment allocation was unblinded.

**Trial registration:**

Current Controlled Trials, ID: ISRCTN11225767. Registered on 3 July 2012;

Clinicaltrials.gov, ID: NCT01658124. Registered on 26 July 2012.

**Electronic supplementary material:**

The online version of this article (10.1186/s13063-019-3561-7) contains supplementary material, which is available to authorized users.

## Background

Acute gastrointestinal (GI) bleeding is a common medical emergency and an important cause of mortality worldwide. Bleeding can occur from the upper or lower GI tract, with upper GI bleeding accounting for most cases. The incidence varies widely depending on the population prevalence of risk factors, with a reported incidence of upper GI bleeding of 50–140 per 100,000 across the US, Europe and Scandinavia [1–9]. The case fatality rate is around 10% for upper GI bleeding [1, 10] and 3% for lower GI bleeding [11]. Despite evidence suggesting improvements in survival in recent decades, the case fatality rate for upper GI bleeding varies from 3 to 15%, with the highest risk of death in patients with upper GI malignancies and varices [1, 3, 4, 8, 10, 12–16]. In addition to cause of bleeding, other factors associated with mortality include older age, signs of shock, severe bleeding, active bleeding, rebleeding and extent of comorbid disease [16–20].

The main causes of GI bleeding are peptic ulcer disease, erosive mucosal disease, oesophageal varices and malignancy [10]. Peptic ulcer disease and erosions due to *Helicobacter pylori* infection and non-steroidal anti-inflammatory drug (NSAID) use are common causes of GI bleeding worldwide [1, 6, 10, 12, 18, 21–25]. Bleeding from gastro-oesophageal varices due to liver cirrhosis is an increasing cause of bleeding in the West, but is also a major cause in parts of South America, Asia, Africa and the Middle East where there is high prevalence of hepatitis or schistosomiasis [26–33]. Symptoms of GI bleeding include haematemesis and coffee ground vomitus, melaena and the passage of fresh red blood in the stool, and clinical signs of shock such as hypotension and tachycardia.

Some patients with GI bleeding initially stop bleeding and have a brief period of haemodynamic stability before starting to bleed again. This phenomenon, known as rebleeding, is common and can affect between 5 and 40% of patients with acute GI bleeding. Rebleeding is associated with a four-fold increased risk of death [10, 11, 16, 17, 34]. Some of the variation in rebleeding rates may be explained by the use of different definitions, including fresh haematemesis or melaena and recurrent hypotension or tachycardia within varying timeframes of the index bleed [18]. The risk of rebleeding is highest in the days immediately after the index bleed and declines rapidly with time [35–37]. The risk factors for rebleeding are related to the lesion responsible for bleeding, but also influenced by age, comorbidity and concomitant medication use. [16, 17].

Tranexamic acid (TXA) reduces clot breakdown by inhibiting the degradation of fibrin by plasmin. It decreases bleeding and the need for blood transfusion in surgery and reduces death due to bleeding in patients with traumatic and postpartum haemorrhage [38–40]. A systematic review and meta-analysis of TXA in patients with upper GI bleeding included eight randomised trials with a total of 1702 patients [41]. Although there was a statistically significant reduction in mortality with TXA (RR 0.60, 95% CI 0.42–0.87; *p* = 0.007) and a non-significant reduction in rebleeding (RR 0.72, 95% CI 0.50–1.03), because of methodological weaknesses in the included trials and the imprecise effect estimates from meta-analyses, the effectiveness and safety of TXA in GI bleeding remains uncertain [41]. Moreover, the included trials were too small to assess the effect of TXA on thromboembolic events. The Haemorrhage ALleviation with Tranexamic acid – Intestinal system (HALT-IT) trial aims to provide reliable evidence about the effects of TXA in acute GI bleeding [42].

## Methods

### Trial design

The HALT-IT trial is an international, randomised, double-blind (participants and trial staff), placebo-controlled trial to quantify the effects of TXA on morbidity and mortality in adults with significant upper or lower GI bleeding.

### Blinding and randomisation

Pfizer Manufacturing, Marketing Authorisation number PL 00057/0952, manufactures the TXA. Torbay and South Devon NHS Foundation Trust, Manufacturing Authorisation number MIA (IMP) 13079, manufactures the placebo (sodium chloride 0.9%). Sharp Clinical Services (UK) Ltd., Manufacturing Authorisation number MIA (IMP) 10284, manufactures the study drug treatment packs containing either the active drug TXA or placebo. The Marketing Authorisation guarantees that the product is manufactured and released in accordance with the UK’s Good Manufacturing Practice (GMP) regulations. Ampoules and packaging are identical in appearance.

An independent statistician from Sealed Envelope Ltd. (UK) generates randomisation codes to be sent to Sharp Clinical Services UK Limited, a GMP-certified clinical trial supplies company that prepares trial treatment packs in accordance with the randomisation list. Sharp Clinical Services conduct the blinding process and first-stage Qualified Person (QP) release, which involves complete removal of the original manufacturer’s label and replacement with the clinical trial label bearing the randomisation number for use as the pack identification. Other pack-label text are identical for TXA and placebo treatments and in compliance with requirements for investigational medicinal products. Sharp Clinical Services UK are also responsible for maintaining the Product Specification File (PSF) until final database lock and unblinding of the trial data. Quality control checks to assure the blinding process are performed on a random samples of final QP released drug packs. High-performance liquid chromatography (HPLC) separation of known TXA is assessed against blinded samples to confirm which ampoule contains the placebo and active treatment. The tested samples are unblinded to assure accuracy of blinding.

The Trial Coordinating Centre (TCC) is responsible for assuring that all relevant approvals are available at the TCC before release of the trial treatment to a site. A separate Manual of Operating Procedures details the drug accountability system. The Investigator’s Brochure details labelling of the trial treatment and other processes for assuring adherence to GMP.

Eligible patients are randomised to receive either TXA or placebo as soon as possible and the study treatment started immediately. The next consecutively numbered treatment pack is taken from a box of eight packs. A fixed loading dosage of 1 g TXA or placebo (sodium chloride 0.9%) is administered, followed by a maintenance dose of 3 g TXA or placebo (sodium chloride 0.9%) infused over 24 h.

### Ethics approval and consent

The trial was approved by the UK NRES Committee East of England (reference number 12/EE/0038), as well as national and local research ethics committees of participating countries outside of the UK.

Acute severe GI bleeding can be a frightening condition for the patient and the ensuing blood loss may have adverse impact on the patient’s mental and emotional state, impairing their decision-making ability. The consent procedures consider this together with the need to randomise and treat urgently. If the patient is fully competent, written consent is sought. If the patient’s capacity is impaired and a personal or professional representative is available, consent is sought from the representative. If neither are able to provide informed consent, consent is waived and the patient is informed about the trial as soon as it is possible.

### Data collection

The entry form (Additional file 1) is used to assess eligibility and collect baseline information. Once a patient has been randomised, the outcome in hospital is collected even if the trial treatment is interrupted or is not actually given. No extra tests are required but a short outcome form (Additional file 1) is completed from the medical records 28 days after randomisation or on discharge from the randomising hospital or on death (whichever occurs first). Any adverse events that become known to the investigator are reported up to 28 days after randomisation.

### Change in primary outcome

**Fig. 1:**
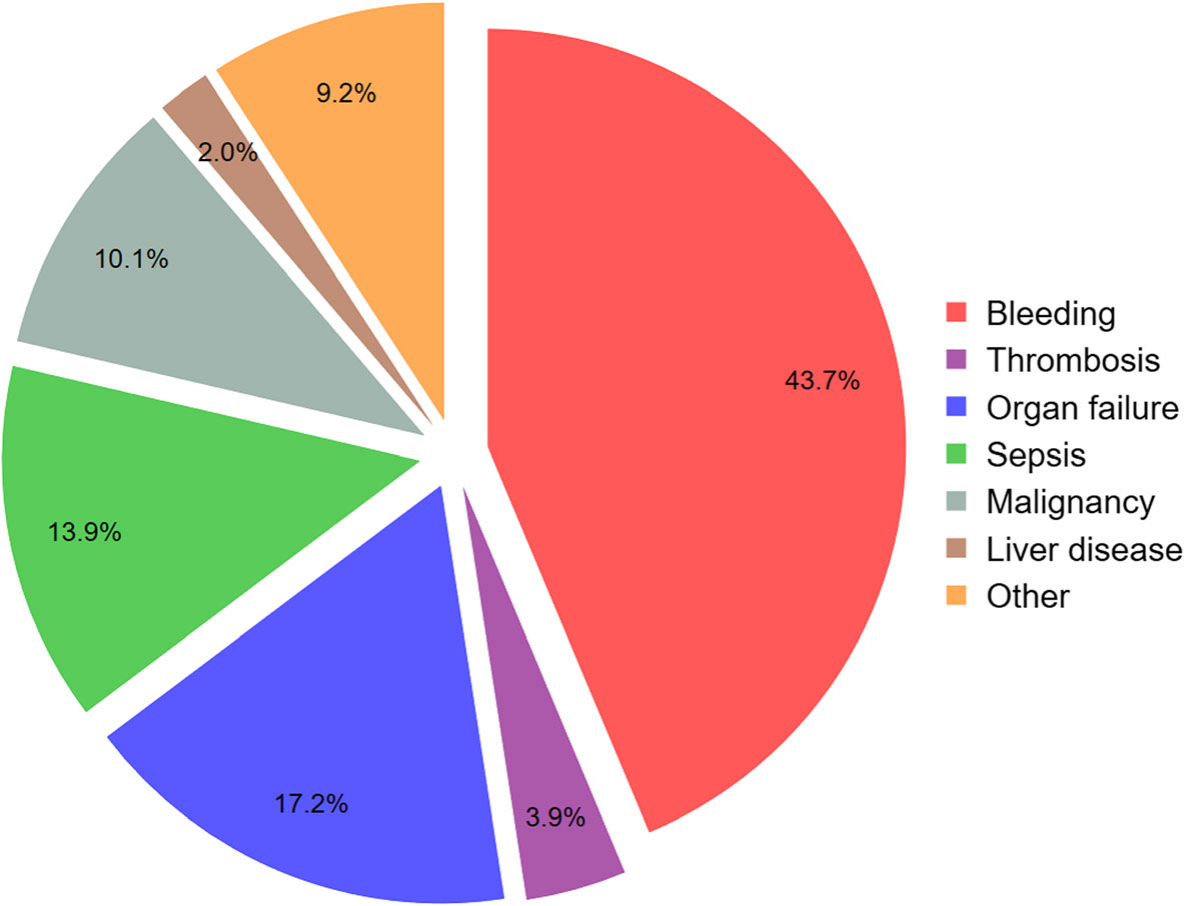
Causes of death in the Haemorrhage ALleviation with Tranexamic acid – Intestinal system (HALT-IT) trial during recruitment (November 2018)

We originally specified all-cause mortality as the primary outcome because we believed that most deaths would be due to bleeding. However, as the trial was underway we observed that over half of all deaths were due to non-bleeding causes such as cancer and sepsis (see Fig. 1). Tranexamic acid reduces bleeding by inhibiting fibrinolysis. Based on this mechanism of action, we do not expect any substantial reduction in non-bleeding deaths. This hypothesis is supported by evidence from trials of TXA in trauma and postpartum haemorrhage [39, 40, 43]. As such, the treatment effect on all-cause mortality will be diluted by non-bleeding causes of death, reducing statistical power [43].

Death due to bleeding is the relevant endpoint for the HALT-IT trial because it has the potential to be reduced by the trial treatment. Fibrinolysis may play an important role in GI bleeding: gastric vein blood samples from patients with peptic ulcers contain high concentrations of plasmin and many patients with acute upper GI bleeding have elevated levels of fibrin degradation products (a biomarker for fibrinolysis) which is associated with worse outcomes [44–46].

Cause of death is assigned by local investigators and a narrative of the events leading to death is reviewed by the principal investigator (who is blind to treatment allocation) and queried as necessary to verify cause of death. Due to the double-blind nature of the trial, the coding of the cause of death cannot be affected by the patient’s randomised group.

We also originally specified that the primary outcome would be measured up to 28 days after randomisation. However, patients receive TXA (or placebo) for their initial bleed but not for rebleeding episodes. Tranexamic acid has a half-life of 2–3 h so 99% will be eliminated within about 2 days of randomisation [47, 48]. We do not expect TXA to reduce deaths from a rebleeding episode for several weeks after the drug has been fully eliminated, therefore the primary outcome will consider early deaths due to bleeding only, defined as those that occur within 5 days of randomisation.

The rationale for refining the primary outcome from all-cause mortality to death due to bleeding was published in October 2018 [43]. The decision was supported by the Trial Steering Committee and was made prior to the end of the trial and prior to unblinding and so was not a data-dependent change.

### Sample size

The sample size calculation for the trial was based on the original primary outcome of all-cause mortality [42]. While the trial was underway, accumulating evidence from other large trials of TXA showed no apparent effect on non-bleeding causes of death [39, 40]. Because a considerable proportion of deaths in the HALT-IT trial are due to non-bleeding causes, the sample size was increased from 8000 to 12,000 to retain sufficient power for all-cause mortality. However, our assumptions were too generous – we assumed that 60% of deaths would be due to bleeding by the end of the trial rather than 40% (assuming a control group event rate of 10%, a study with 12,000 participants would have over 80% power to detect a 15% (RR = 0.6 × 0.75 + 0.4 × 1.0 = 0.85) reduction in all-cause mortality). Based on the refined primary outcome, assuming a cumulative incidence of death due to bleeding of 4%, a study with 12,000 patients will have about 85% power (two-sided alpha = 5%) to detect a clinically important 25% relative reduction in death due to bleeding from 4 to 3%. Loss to follow-up is expected to be less than 1% (it was 0.16% in the World Maternal Antifibrinolytic (WOMAN) trial) so was not taken into consideration when calculating the sample size. This power calculation is based on the primary analysis and refers to the unadjusted chi-squared test.

## Trial population

### Eligibility

Patients with significant GI bleeding to whom the uncertainty principle applies are eligible. Specifically, a patient can be enrolled if the responsible clinician is substantially uncertain as to whether the trial treatment is appropriate for that particular patient. Significant bleeding is diagnosed clinically and implies a risk of bleeding to death. Patients with significant bleeding may include those with hypotension, tachycardia, signs of shock, or those needing urgent transfusion, endoscopy or surgery. Patients with a clear indication (e.g. traumatic haemorrhage) or contraindication (e.g. history of convulsions, thromboembolic disease) for TXA are excluded.

### Recruitment, withdrawal and loss to follow-up

We will display the flow of study participants using a Consolidated Standards of Reporting Trials (CONSORT) Diagram (see Additional file 1: Figure S1). For each trial arm, we will present the total number randomised, the number with baseline data, the number lost to follow-up, the number who withdrew consent, and the number of participants with outcome data.

### Baseline patient characteristics

We collect data on the following baseline characteristics: age, biological sex, time from onset of GI bleeding symptoms to randomisation, suspected location of bleeding, clinical symptoms (e.g. haematemesis, melaena), suspected variceal bleeding, systolic blood pressure (SBP) , heart rate (HR), signs of shock, suspected active bleeding, major comorbidities, anticoagulation therapy and type of admission. We will present the distribution of baseline characteristics (*n* and %) in the treatment and placebo groups to check that randomisation was successful in producing similar groups (see Additional file 1: Table S1).

## Analysis

### Primary analysis

The main analyses will compare those allocated TXA with those allocated placebo on a modified intention-to-treat basis, excluding patients who received neither dose of the allocated trial treatment. We will present the results as effect estimates (relative risks) with a measure of precision (95% confidence intervals) (see Additional file 1: Table S2). Additionally, we will present results of the primary analysis adjusted for all baseline covariates. If baseline covariates are associated with the outcome, adjusting for any chance imbalances in baseline risk will increase statistical power. We will not present risk differences because they are not a generalisable measure of the treatment effect and are dependent on baseline risk. The effect of TXA will also be examined graphically using cumulative incidence curves presented with their associated hazard ratios and log-rank *p* values (see Additional file 1: Figure S2) [49]. The effects of TXA on death due to bleeding in the HALT-IT trial will be set in the context of other trials of TXA for acute severe haemorrhage (the CRASH-2 and WOMAN trials).

### Primary outcome

Death due to bleeding within 5 days of randomisation is the primary outcome. As outlined in the section ‘Change in primary outcome’ above, cause of death is assigned by local investigators who provide a narrative of the events leading to death. The cause of death narratives are reviewed by the principal investigator (who is blind to treatment allocation) and queried if more information is needed to confirm whether death is due to bleeding or another cause. Furthermore, due to double-blind nature of the trial, the coding of the cause of death cannot be affected by the patient’s randomised group. For more details, please see accompanying information in the section ‘Change of primary outcome’.

### Secondary outcomes

We will assess the effect of TXA on the following secondary outcomes. Unadjusted analyses will be presented in the main text and although we do not expect any baseline imbalances, to complement the unadjusted analyses and potentially increase statistical power (if covariates are associated with the outcome) we will present results of the analyses adjusted for all baseline covariates in an appendix.

#### Rebleeding

Rebleeding generally occurs in approximately 10–25% of patients with acute GI haemorrhage and is associated with an increased risk of death due to bleeding [50]. A clinical diagnosis of rebleeding is made by the treating clinician based on the presence of any of the following criteria, as defined in a data collection guide. These criteria for rebleeding were recommended by a methodological framework for trials in GI bleeding following an international consensus conference [51]:

- Haematemesis or bloody nasogastric aspirate > 6 h after endoscopy
- Melaena after normalisation of stool colour
- Haematochezia after normalisation of stool colour or after melaena
- Development of tachycardia (HR > 110 beats per min) or hypotension (SBP ≤ 90 mmHg) after ≥ 1 h of haemodynamic stability (i.e. no tachycardia or hypotension) in the absence of an alternative explanation for haemodynamic instability such as sepsis, cardiogenic shock, or medication
- Haemoglobin drop of > 2 g/dl after two consecutive stable values (< 0.5 g/dl decrease) ≥3 h apart
- Tachycardia or hypotension that does not resolve within 8 h after index endoscopy despite appropriate resuscitation (in the absence of an alternative explanation) associated with persistent melaena or haematochezia
- Persistently dropping haemoglobin of > 3 g/dl in 24 h associated with persistent melaena or haematochezia

It should be noted that patients may continue to have haemodynamic instability, falling haemoglobin levels or persistent melaena or rectal bleeding for hours and even days after bleeding has stopped, making these patients difficult to categorise; however, these criteria are more likely to indicate rebleeding than equilibration [51].

Most rebleeding tends to occur within 5 days of the index bleed [35–37]. We believe that TXA will be most effective at reducing the risk of rebleeding soon after the index bleed when blood plasma concentrations of the drug are above the level needed to inhibit fibrinolysis [52]. To assess whether TXA reduces rebleeding, we will analyse the effect on early rebleeding within 5 days of randomisation (see Additional file 1: Table S2).

Although rebleeding is most common within the first 5 days after the index bleed, TXA will have been metabolised within about 2 days of randomisation, with the blood plasma concentration falling below the level needed to inhibit fibrinolysis within around 24 h. As such, we will examine the effect on rebleeding within 24 h of randomisation. We hypothesise that TXA will be less effective for late rebleeding occurring days or weeks after the drug has been eliminated. To investigate this we will assess the effect of TXA on rebleeding within 28 days (see Additional file 1: Table S2). If our hypothesis is correct, the inclusion of late rebleeding events should dilute the treatment effect.

#### Death due to bleeding within 24 h and 28 days

Tranexamic acid will be eliminated within about 2 days of randomisation, with blood plasma levels falling below those needed to inhibit fibrinolysis within around 24 h. Furthermore, patients with acute GI haemorrhage bleed to death quickly, with many deaths due to bleeding occurring within the first day. Evidence from other trials suggests that this is where the greatest treatment benefit will be observed. As such, we will analyse the effect of TXA on deaths due to bleeding within 24 h of randomisation. Conversely, because there may be a weaker treatment effect on late deaths due to bleeding that occur several days or weeks after randomisation, we will also analyse the effect on death due to bleeding within 28 days of randomisation (see Additional file 1: Table S2). We expect to observe a smaller treatment effect when including late bleeding deaths due to dilution towards the null.

#### Mortality

We will analyse the effect of TXA on all-cause and cause-specific mortality at 28 days. Specific causes of death to be analysed include death due to bleeding, thrombosis, organ failure, pneumonia, sepsis, malignancy and other causes (see Additional file 1: Table S3). We will also examine the temporal distribution of causes of death by days since randomisation using a frequency bar chart (see Additional file 1: Figure S3). Based on its mechanism of action and data from large randomised trials, we do not expect TXA to reduce deaths from non-bleeding causes like cancer or sepsis or to reduce late deaths from bleeding.

#### Endoscopic, radiological and surgical procedures for GI bleeding

We will assess the effect of TXA on diagnostic and therapeutic endoscopic and radiological procedures and surgical interventions (see Additional file 1: Table S5). It remains unclear whether TXA reduces the need for surgery in GI bleeding [41]. In large trials of TXA for postpartum and traumatic haemorrhage, there was no evidence of an effect on surgical interventions except for laparotomy for bleeding [39, 40]. If TXA reduces GI bleeding, it has the potential to reduce the need for some endoscopic, radiological and surgical procedures. While we do not expect TXA to influence diagnostic endoscopic and radiological procedures planned around the time of hospital admission and randomisation, there is potential to reduce the need for diagnostic procedures planned after resuscitation, and, therefore, after randomisation [43]. Similarly, therapeutic procedures and surgical interventions planned and undertaken after diagnosis also have the potential to be influenced by TXA. It is not possible to look at procedures by time as this information was not recorded, and although type of procedure can be used as a rough indication of timing, therapeutic or surgical procedures planned around the time of randomisation could still dilute the effect estimates towards the null.

#### Blood transfusion

Since blood transfusion is mostly determined by blood loss prior to randomisation, we do not expect to see a marked reduction in the need for blood transfusion with the use of TXA [43]. Major haemorrhage protocols dictate the type and volume of blood components that patients receive based on presenting clinical signs such as blood pressure and estimated blood loss. Furthermore, survivor bias could lead to higher transfusion rates in the TXA group. In keeping with this, a systematic review of TXA for GI bleeding found no reduction in transfusion [41]. Although TXA has the potential to reduce transfusion for blood lost after randomisation, e.g. after rebleeding, we did not collect data on date and time of transfusion. Any effect on late transfusions is likely to be obscured by early transfusions for blood lost pre-randomisation. We will assess the effect of TXA on the use of whole blood or packed red cells, frozen plasma and platelets comparing the frequency of transfusion and the mean number of (adult-equivalent) units transfused (see Additional file 1: Table S5).

#### Thromboembolic events

An individual patient data meta-analysis of the WOMAN and CRASH-2 trials found evidence of a reduction in myocardial infarction with TXA (OR = 0·64, 95% CI 0·43–0·97; *p* = 0·037) and no evidence of an increased risk of fatal vascular occlusive events (OR 0·73, 95% CI 0·49–1·09; *p* = 0·120) or other non-fatal events [53]. While this finding is reassuring, we cannot exclude the possibility of some increased risk with TXA, particularly as patients with GI bleeding are older than those with traumatic or postpartum haemorrhage and many have multiple comorbidities. Older age is associated with a pro-coagulation haemostatic profile including elevated fibrinogen and plasminogen activator inhibitor 1 and reduced clotting time [54–56]. A systematic review of TXA for the treatment of upper GI bleeding found no evidence for a difference in the risk of thromboembolic events but lacked power [41]. We will examine the effect of TXA on fatal and non-fatal pulmonary embolism, deep vein thrombosis, stroke and myocardial infarction (see Additional file 1: Table S6).

#### Complications

We will analyse the effect of TXA on renal, hepatic and respiratory failure, cardiac events, sepsis, pneumonia and seizures (see Additional file 1: Table S6). If TXA reduces death due to bleeding, patients in the tranexamic group will survive for longer on average and may, therefore, be at greater risk of complications such as sepsis, pneumonia and organ failure. Generally, death due to bleeding tends to occur soon after bleeding onset whereas infections and organ failure take several days to occur. On the other hand, if TXA reduces bleeding it may reduce liver failure because bleeding can lead to the deterioration of liver function. Although there is evidence that high-dose TXA can cause seizures, we do not expect to see an increase in seizures with the low dose given in the trial.

#### Self-care capacity

Patients self-care capacity will be measured using the Katz Index of Independence in Activities of Daily Living (Katz ADL) [57]. Participants’ performance in six functions (bathing, dressing, toileting, transferring, continence and feeding) is assessed at the time of discharge from the randomising hospital or in hospital 28 days after randomisation. A score of 1 is assigned to each function the individual can perform independently and they are summed to produce a total score. A score of 6 suggests full function, 4 suggests moderate impairment, and 2 or less suggests severe functional impairment. We expect that reduced blood loss in patients who receive TXA will result in less functional impairment. That said, it is possible that patients in the treatment group will be discharged faster which could mask improvements in self-care capacity at the time of discharge. To assess this hypothesis we will compare the difference in mean Katz ADL score in survivors in the TXA and placebo groups as well as the proportion of patients with no impairment (6), mild to moderate impairment (3–5) or severe impairment (0–2), (see Additional file 1: Table S6).

#### Days spent in the intensive care or high-dependency unit

We will analyse the effect of TXA on number of days spent in the intensive care unit (ICU) or high-dependency unit (HDU). We will compare the difference in mean number of days spent in the ICU or HDU in the TXA and placebo groups (see Additional file 1: Table S6). Because beds in these units can be limited, we may not see an effect on this outcome measure.

#### Adverse events

Data on the number of adverse events (AEs), serious adverse events (SAEs) and suspected unexpected serious adverse reactions (SUSARs) reported up to 28 days after randomisation will be presented. We will present a summary table in an Additional file 1 to describe the type of AE, Medical Dictionary for Regulatory Activities (MedDRA) preferred term (PT), MedDRA system organ class (SOC) and the number of occurrences and outcomes (completely recovered, recovered with sequelae, or died) in the TXA and placebo groups. With events grouped by MedDRA SOC, we will compare the frequency of events between trial arms using an unadjusted modified Poisson regression model (see Additional file 1: Table S7). AEs with evidence that they may be increased by TXA (i.e. seizures and thromboembolic events), will be analysed on an individual basis as well as recurrent episodes of GI bleeding reported as AEs.

### Subgroup analyses

We will conduct the following subgroup analyses for the primary outcome of death due to bleeding: time to treatment, location of bleeding, cause of bleeding and clinical Rockall score. We will fit interaction terms with randomised group in a Poisson regression model with robust error variance from the sandwich estimator [58]. Interaction tests (the Wald test) will be used to explore whether the effect of treatment (if any) differs across these subgroups. Results will be presented as unadjusted and adjusted effect estimates with a measure of precision (95% confidence intervals) and *p* value for the test for interaction (see Additional file 1: Table S4). Except for time to treatment, statistically significant heterogeneity between subgroups is required, as determined by the test for interaction *p* value, and not just statistical significance of a result in a specific subgroup [59].

**Fig. 2:**
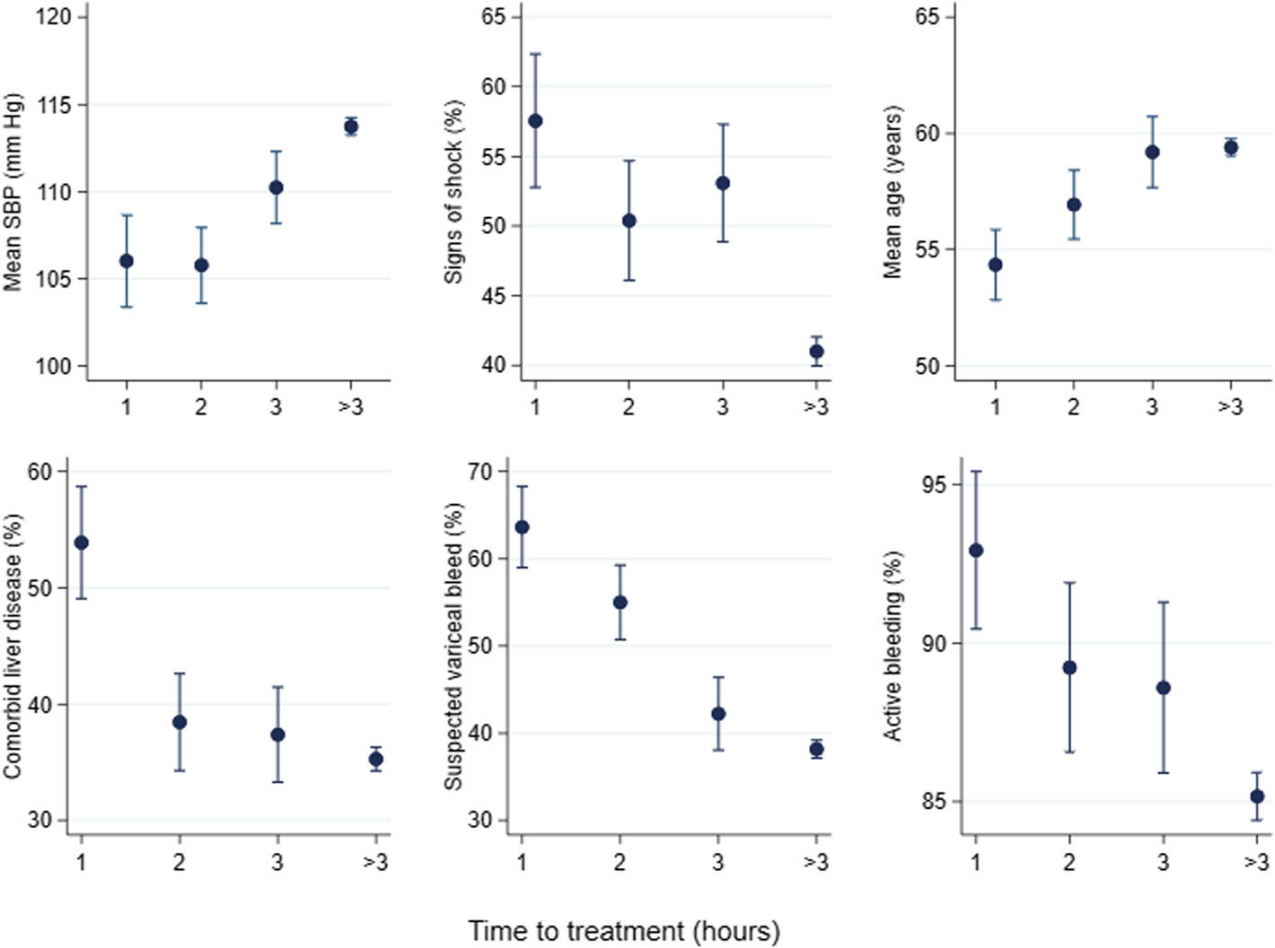
Potential confounding factors in the subgroup analysis of time to treatment

Although treatment group is randomised within subgroups, the factors defining subgroups are not randomised. Several baseline characteristics are associated with the subgroup variables. For example, early treatment is correlated with bleed characteristics and patient characteristics (see Fig. 2), some of which confer a higher clinical Rockall score, suggesting that patients with more severe bleeding are treated earlier. Since these factors are also associated with mortality, they could potentially confound the interaction between time to treatment and the treatment effect.

If TXA is shown to be effective and the treatment effect varies by time to treatment, there is potential to intervene on time to treatment in order to increase the treatment effect. Although we cannot intervene on location of bleeding, cause of bleeding or clinical Rockall score, we are interested in ascertaining causal interaction of these factors with the treatment effect rather than simply assessing effect heterogeneity. As such, we will adjust all subgroup analyses for potential confounders [60]. Selection of potential confounders is based upon review of unblinded data within the trial to date in order to identify prognostic baseline characteristics that are associated with the subgroup variables. Potential confounders include age, time to treatment, SBP, HR, signs of shock, location of bleeding, suspected active bleeding, comorbid liver disease and suspected variceal bleeding. Signs of shock may be collinear with HR or blood pressure, and suspected variceal bleeding may be collinear with comorbid liver disease – if so, signs of shock and suspected variceal bleeding will not be included in the models. The final models remain to be determined because the outcome of interest is the treatment effect and the association between the potential confounders and the treatment effect cannot be assessed before unblinding.

#### Time to treatment (≤ 3 h, > 3 h)

Trials of TXA in traumatic and postpartum haemorrhage provide evidence that early treatment (within 3 h of bleeding onset) confers the most benefit, while late treatment is ineffective [39, 53, 61]. As such, we plan to conduct a subgroup analysis of the treatment effect stratified by time to treatment. Patients with GI bleeding may not experience symptoms immediately so time of symptom onset may not accurately reflect time of bleeding onset. Time to treatment may, therefore, be underestimated. Because few patients are treated early (within 3 h), there may be low power to detect an interaction if one exists. As such, we will analyse time to treatment as both a categorical (≤ 3 h, > 3 h) and continuous variable because the latter will preserve more information so should have more power. However, a limitation of modelling time to treatment as a continuous variable is the need to specify the form of the association. To assess non-linearities, we will fit a logistic regression model and use a likelihood ratio test. Any differences between the two approaches will be noted.

There is strong prior evidence to expect a time-to-treatment interaction, with early treatment conferring a greater benefit and late treatment being ineffective and possible even harmful [53, 61]. As such, for the subgroup analysis of time to treatment we do not require as strong evidence against the null hypothesis of homogeneity as we might usually require. Most trials lack power to detect heterogeneity in treatment effects and the lack of a statistically significant interaction does not mean that the overall treatment effect applies to all patients. Due to prior evidence that early treatment is more effective, we will consider the time to treatment subgroup analysis in the context of the existing data (in particular data from the CRASH-2 and WOMAN trials) on the time-to-treatment interaction and will rely more on scientific judgment than on statistical tests.

#### Location of bleeding (upper GI, lower GI)

We will examine the effect of TXA on death due to bleeding stratified by location (upper versus lower GI). Evidence suggests the rates of rebleeding and mortality after upper and lower GI bleeding are similar [34], and there is no reason to expect the effect of TXA to vary substantially by location of bleeding in the GI tract. Unless there is strong evidence against the null hypothesis of homogeneity of effects (i.e. *p* < 0.01), the overall relative risk will be considered the most reliable guide to the approximate treatment effect in all patients.

#### Suspected variceal bleeding and comorbid liver disease (yes, no/unknown)

Outcomes in acute GI bleeding vary by cause of haemorrhage. Variceal bleeding is associated with the highest risk of rebleeding and death. Oesophageal varices are dilated submucosal veins that usually develop because of portal hypertension, often due to cirrhosis. Haemostasis is disturbed in patients with liver disease because many of the pro- and anti-coagulation factors and components of the fibrinolytic system are produced by hepatic parenchymal cells in the liver, although the overall sum of effects are debated [62–64]. Any resulting imbalance in coagulation or fibrinolysis may alter the antifibrinolytic activity of TXA; however, the direction of this potential effect remains to be determined. We will examine the effects of TXA on death due to bleeding in patients with suspected variceal bleeding and comorbid liver disease compared to other or unknown causes of bleeding. Unless there is strong evidence against the null hypothesis of homogeneity of effects (i.e. *p* < 0.01), the overall relative risk will be considered the most appropriate measure of effect.

#### Clinical Rockall score (1–2, 3–4, 5–7)

We will assess the effect of TXA stratified by the clinical (pre-endoscopy) Rockall score, a widely used risk scoring system for GI bleeding. The score is derived from age, comorbidities, signs of shock, HR and SBP, all of which are independent predictors of mortality. Although originally developed for upper GI bleeding [17], the Rockall score has also been shown to be predictive of mortality in lower GI bleeding [34]. We do not expect the treatment effect to vary by Rockall score. Unless there is strong evidence of an interaction (*p* < 0.01), we will present the overall relative risk as the most appropriate measure of effect.

### Missing data

Based on the data collected to date, we expect loss to follow-up to be minimal (i.e. less than 1% missing data on the primary outcome). Any missing values will be reported but not imputed.

## Other analyses to be reported in separate publications

### Survival analysis to investigate the timing and duration of the treatment effect

We will conduct a survival analysis to explore the effect of TXA on rebleeding and death due to bleeding in more detail. In large trials of TXA for traumatic (CRASH-2) and postpartum haemorrhage (WOMAN), there were few late-bleeding-related events. The precise timing and duration of TXA’s antifibrinolytic effect remain to be determined. For example, it is unclear whether the treatment effect persists after the drug has been eliminated. Bleeding-related events occur later in acute GI bleeding, partly due to rebleeding, so the HALT-IT trial presents a unique opportunity to investigate this question.

We will report the median survival time and the cumulative incidence in the treatment and placebo groups, and model the treatment effect. Cox proportional hazards modelling assumes the hazards in the treatment and placebo groups are proportional over time. This assumption may be invalid if the antifibrinolytic effect of TXA declines over time as the drug is metabolised. We will formally assess this using the Royston-Palmer test for proportional hazards – a combined test with increased power when an early treatment effect is present [65]. If the treatment effect on death due to bleeding and rebleeding appears to change with time (non-proportional hazards), we will examine this in detail using various methods. We will estimate average cumulative hazard ratios for increasingly longer periods of follow-up. This method is preferred to period-specific hazard ratios, which can be susceptible to selection bias [66]. Nevertheless, we will also use Lexis expansion to calculate period-specific hazard ratios and test for interactions between treatment group and period. If we are able to identify the average duration of the treatment effect, we will examine whether this varies by baseline characteristics including time to treatment, bleeding severity, cause of bleeding and age. We will also assess how the treatment effect changes with time by including a time-by-treatment interaction term in the model. Residual methods will be used to test the assumption of linear time (first order trend) by plotting Martingale residuals against continuous covariates.

Death due to bleeding is a competing risk for non-bleeding causes of death and vice versa. Death is also a competing risk for rebleeding. We will estimate the treatment effect using a proportional cause-specific hazards model in which competing events are censored. The proportional cause-specific hazards model is preferred for aetiological research; however, both the cause-specific hazard and cumulative incidence can provide insights into a treatment’s effects [67, 68]. As such, a subdistribution hazards model and Gray’s test for comparing cumulative incidence functions will be presented as a supplementary analysis [69, 70]. Risk of rebleeding is highest immediately after the index bleed, death is a competing risk for rebleeding and some patients may experience more than one episode during the follow-up period. A survival analysis of the effect of TXA on rebleeding will take into account timing of events and competing risks.

### Cost effectiveness analysis

If the trial demonstrates that TXA is an effective treatment for GI bleeding, we will conduct an economic evaluation to determine cost-effectiveness. Broadly speaking the methods will mirror those used by Li et al. who assessed the cost-effectiveness of TXA for the treatment of women with postpartum haemorrhage [71].

The analysis will compare TXA against clinical practice without TXA. A cost-utility analysis will be performed from a health services cost perspective with outcomes expressed as Quality-adjusted Life Years (QALYs). The analyses will be performed separately for a set of different countries, depending on where the majority of people have been recruited, but is likely to include at least the UK and Pakistan. A decision model will be used to extrapolate results from the trial into the longer term. Resource data, such as drugs and length of inpatient stay, are collected as part of the trial and will be analysed accordingly. Both deterministic and probabilistic sensitivity analysis will be undertaken. Results will also be presented by subgroups if considered appropriate.

### Impact of baseline risk on treatment effectiveness

To assess whether the effect of TXA on death due to bleeding varies by baseline risk we will build a prognostic model using baseline characteristics identified as important predictors of death due to bleeding. Prognostic factors include age, SBP, HR, suspected location of bleeding, haemetamesis/coffee ground vomitus, suspected variceal bleeding, suspected active bleeding, comorbidities and country. The prognostic model will then be used to stratify patients by risk of mortality and stratum-specific effect estimates (relative risk) and 95% confidence intervals will be calculated. We do not expect the treatment effect to vary by baseline risk. Unless there is strong evidence against the null hypothesis of homogeneity of effects (*p* < 0.01), the overall relative risk will be considered the most reliable guide to the approximate treatment effect in all patients.

### Adjustment for baseline risk

Due to the large size of the HALT-IT trial, baseline characteristics should be well balanced between the treatment and placebo groups so that any difference in outcomes is due to the treatment. There is still a small possibility, however, that some imbalance in baseline risk may have arisen by chance. If prognostic factors are distributed differently across the treatment and placebo groups, this could bias the treatment effect. To investigate this hypothesis, we will conduct an analysis of the treatment effect on death due to bleeding adjusted for baseline risk. Patients will be stratified by risk deciles based on the predicted probability of death due to bleeding and a pooled effect estimate (relative risk) will be calculated using inverse variance weighting. This will provide an estimate of the treatment effect where both groups have equal baseline risks.

### Centre and country effects

Centre- and country-level characteristics can influence patient outcomes. Differences in outcome may be related to resource availability or clinical practice. To explore between-country differences we will present a graph showing the number of patients and bleeding deaths by country and will use multivariable regression modelling to examine the treatment effect by country, including an interaction term between country and treatment. We will not adjust for clustering as we expect the effects of clustering to be small. Because we aim to understand any between-country differences in the treatment effect, we will adjust for potential confounders including age, SBP, HR, comorbidities, location of bleeding, suspected variceal bleeding, suspected active bleeding and time to treatment. A comparison between low-, middle- and high-income countries will be included using the World Bank country groupings by income. We do not expect the effect of TXA on the risk of death due to bleeding to vary by country, even though the absolute risk will vary due to between-country differences in patient populations. Countries recruiting fewer than 100 patients will be omitted from the analysis as necessary.

Between-centre differences in outcome may also influence the estimation of the treatment effect. We will first use a mixed-effects regression model using restricted maximum likelihood estimation to examine whether there are differences in death due to bleeding between centres. Results will be presented in the form of a forest plot. Prognostic patient characteristics (age, SBP, HR, comorbidities, location of bleeding, suspected variceal bleeding, suspected active bleeding), treatment group and time to treatment will be adjusted for. To take into account country-level effects we will also consider between-centre differences in outcome adjusted for country. We will then use mixed-effects regression to estimate the treatment effect before and after accounting for between-centre differences, assuming a constant treatment effect across centres. To assess whether the treatment effect differs by centre, we will fit a model with an interaction term between centre and treatment.

## Data monitoring

The progress of the HALT-IT trial, including recruitment, data quality, outcomes and safety data, are reviewed by an independent Data Monitoring Committee, which can decide to reveal unblinded results to the Trial Steering Committee. To date, four interim analyses have been conducted.

## Data sharing

To maximise data utilisation and improve patient care, the trial data will be made available via our online data-sharing portal – The Free Bank of Injury and Emergency Research Data (freeBIRD) (https://ctu-app.lshtm.ac.uk/freebird/) – once primary and secondary analyses have been published.

## Trial status

The study has been actively recruiting since July 2013. End of recruitment is planned for 31 May 2019, with end of follow-up expected on 30 June 2019. Further information is available at http://haltit.Lshtm.ac.uk/.

## Discussion

We present our plan for the statistical analysis of the HALT-IT trial prior to the end of recruitment, database lock and unblinding in order to avoid data-dependent analyses. We set out a-priori hypotheses and propose ways to test these. We also provide the rationale for changing the primary outcome from all-cause mortality to death due to bleeding within 5 days of randomisation.

## Additional file

Additional file 1 **Figure S1** Trial profile. **Figure S2** Cumulative percentage of death due to bleeding in the tranexamic acid and placebo groups. **Figure S3** Distribution of cause of death by days since randomisation. **Table S1** Baseline characteristics of participants prior to randomisation. **Table S2** Death due to bleeding and rebleeding. **Table S3** Other causes of death and all-cause mortality. **Table S4** Death due to bleeding by subgroups. **Table S5** Need for surgical, endoscopic and radiological interventions and blood transfusion. **Table S6** Thromboembolic events, complications and self-care capacity. **Table S7** Adverse events. (DOC 355 kb)

## Data Availability

The datasets generated and/or analysed during the current study are not yet publicly available because the trial is ongoing. Once recruitment has stopped and after publication of the planned primary and secondary analyses, the trial data will be made available via our data-sharing portal, The Free Bank of Injury and Emergency Research Data (freeBIRD) website at https://ctu-app.lshtm.ac.uk/freebird/.

## Abbreviations

AE: Adverse event
CRASH-2: Clinical Randomisation of an Antifibrinolytic in Significant Haemorrhage
GI: Gastrointestinal
GMP: Good Manufacturing Practice
HALT-IT: Haemorrhage ALleviation with Tranexamic acid – Intestinal system
HDU: High-dependency unit
HPLC: High-performance liquid chromatography
HR: Heart rate
ICU: Intensive care unit
Katz ADL: Katz Index of Independence in Activities of Daily Living
MedDRA PT: Medical Dictionary for Regulatory Activities Preferred Term
MedDRA SOC: Medical Dictionary for Regulatory Activities system organ class
MedDRA: Medical Dictionary for Regulatory Activities
PSF: Product Specification File
QALYs: Quality-adjusted Life Years
QP: Qualified Person
SAE: Serious adverse event
SBP: Systolic blood pressure
SUSAR: Suspected unexpected serious adverse reaction
TCC: Trial Coordinating Centre
TXA: Tranexamic acid
UK: United Kingdom
US: United States
WOMAN: World Maternal Antifibrinolytic
