# Supplementary material for: Tranexamic acid for acute gastrointestinal bleeding (the HALT-IT trial): statistical analysis plan for an international, randomised, double-blind, placebo-controlled trial
Source: Trials. 2019 Jul 30;20:467. doi: 10.1186/s13063-019-3561-7 (PMC6668177; doi:10.1186/s13063-019-3561-7)
Supplement: Supplementary file 1 — Figure S1 Trial profile. Figure S2 Cumulative percentage of death due to bleeding in the tranexamic acid and placebo groups. Figure S3 Distribution of cause of death by days since randomisation. Table S1 Baseline characteristics of participants prior to randomisation. Table S2 Death due to bleeding and rebleeding. Table S3 Other causes of death and all-cause mortality. Table S4 Death due to bleeding by subgroups. Table S5 Need for surgical, endoscopic and radiological interventions and blood transfusion. Table S6 Thromboembolic events, complications and self-care capacity. Table S7 Adverse events. (DOC 355 kb) [file 13063_2019_3561_MOESM1_ESM.doc]

**Additional file 1: HALT-IT Statistical Analysis Plan Shell Tables**

Figure S1: **Trial profile**

|  | Randomised(n = 12 000) |  |
| --- | --- | --- |
| TXA (n = 6 000) | Treatment allocation | Placebo (n = 6 000) |
|  |  |  |
| n = NN | Withdrawal after randomisation | n = NN |
|  |  |  |
| Baseline data (n = N NNN)  Received allocated dose 1  (n = N NNN)  Received allocated dose 2  (n = N NNN) | Allocation | Baseline data (n = N NNN)  Received allocated dose 1  (n = N NNN)  Received allocated dose 2  (n = N NNN) |
|  |  |  |
| No follow-up† (n = NN) | Follow-up | No follow-up† (n = NN) |
|  |  |  |
| Deviation/violation of protocol[~reasons] (n = NN)Analysed (n = N NNN) | Analysis | Deviation/violation of protocol[~reasons] (n = NN) Analysed (n = N NNN) |

† No follow-up relates to those patients where there is no information on the primary endpoint

Table S1: **Baseline characteristics of participants prior to randomisation**

| **Baseline characteristics** | **TXA** | | **Placebo** | |
| --- | --- | --- | --- | --- |
|  | (n = 6 000) | | (n = 6 000) | |
|  | n | (%) | n | (%) |
| **Age** |  |  |  |  |
| <40 |  |  |  |  |
| 40-59 |  |  |  |  |
| 60-79 |  |  |  |  |
| 80+ |  |  |  |  |
| **Sex** |  |  |  |  |
| Female |  |  |  |  |
| Male |  |  |  |  |
| **Time from symptom onset to randomisation (hours)** |  |  |  |  |
| <=3 |  |  |  |  |
| >3-<=8 |  |  |  |  |
| >8 |  |  |  |  |
| **Suspected location of bleeding** |  |  |  |  |
| Lower |  |  |  |  |
| Upper |  |  |  |  |
| **Haematemisis/coffee ground vomitus** |  |  |  |  |
| No |  |  |  |  |
| Yes |  |  |  |  |
| **Melaena/fresh blood per rectum** |  |  |  |  |
| No |  |  |  |  |
| Yes |  |  |  |  |
| **Suspected variceal bleeding** |  |  |  |  |
| No |  |  |  |  |
| Yes |  |  |  |  |
| **Suspected active bleeding** |  |  |  |  |
| No |  |  |  |  |
| Yes |  |  |  |  |
| **Major comorbidities** |  |  |  |  |
| Cardiovascular |  |  |  |  |
| Respiratory |  |  |  |  |
| Liver |  |  |  |  |
| Renal |  |  |  |  |
| Malignancy |  |  |  |  |
| Other |  |  |  |  |
| **Systolic blood pressure** |  |  |  |  |
| >=90 |  |  |  |  |
| 76-89 |  |  |  |  |
| <=75 |  |  |  |  |
| Missing |  |  |  |  |
| **Heart rate** |  |  |  |  |
| <77 |  |  |  |  |
| 77-91 |  |  |  |  |
| 92-107 |  |  |  |  |
| >107 |  |  |  |  |
| Missing |  |  |  |  |
| **Signs of shock** |  |  |  |  |
| No |  |  |  |  |
| Yes |  |  |  |  |
|  |  |  |  |  |
| **Bleed severity (clinical Rockall score)** |  |  |  |  |
| 1-2 |  |  |  |  |
| 3-4 |  |  |  |  |
| 5-7 |  |  |  |  |
| **On anticoagulants** |  |  |  |  |
| No |  |  |  |  |
| Yes |  |  |  |  |
| Unknown |  |  |  |  |
| **Emergency admission** |  |  |  |  |
| Yes |  |  |  |  |
| No |  |  |  |  |

Table S2: **Death due to bleeding and rebleeding**

| Outcome | TXA  (n = 6 000) | Placebo  (n = 6 000) | Risk ratio  (95% CI) |
| --- | --- | --- | --- |
| Death due to bleeding within 24 h | NN (X%) | NN (X%) | X.XX (X.XX­–X.XX) |
|  |  |  |  |
| Death due to bleeding within 5 days | NN (X%) | NN (X%) | X.XX (X.XX­–X.XX) |
|  |  |  |  |
| Death due to bleeding within 28 days | NN (X%) | NN (X%) | X.XX (X.XX­–X.XX) |
|  |  |  |  |
| Rebleeding within 24 h | NN (X%) | NN (X%) | X.XX (X.XX­–X.XX) |
| Rebleeding within 5 days | NN (X%) | NN (X%) | X.XX (X.XX­–X.XX) |
|  |  |  |  |
| Rebleeding within 28 days | NN (X%) | NN (X%) | X.XX (X.XX­–X.XX) |
|  |  |  |  |
|  |  |  |  |

† P-values from unadjusted modified Poisson regression model

Figure S2: **Cumulative percentage of death due to bleeding in the tranexamic acid and placebo groups**

**
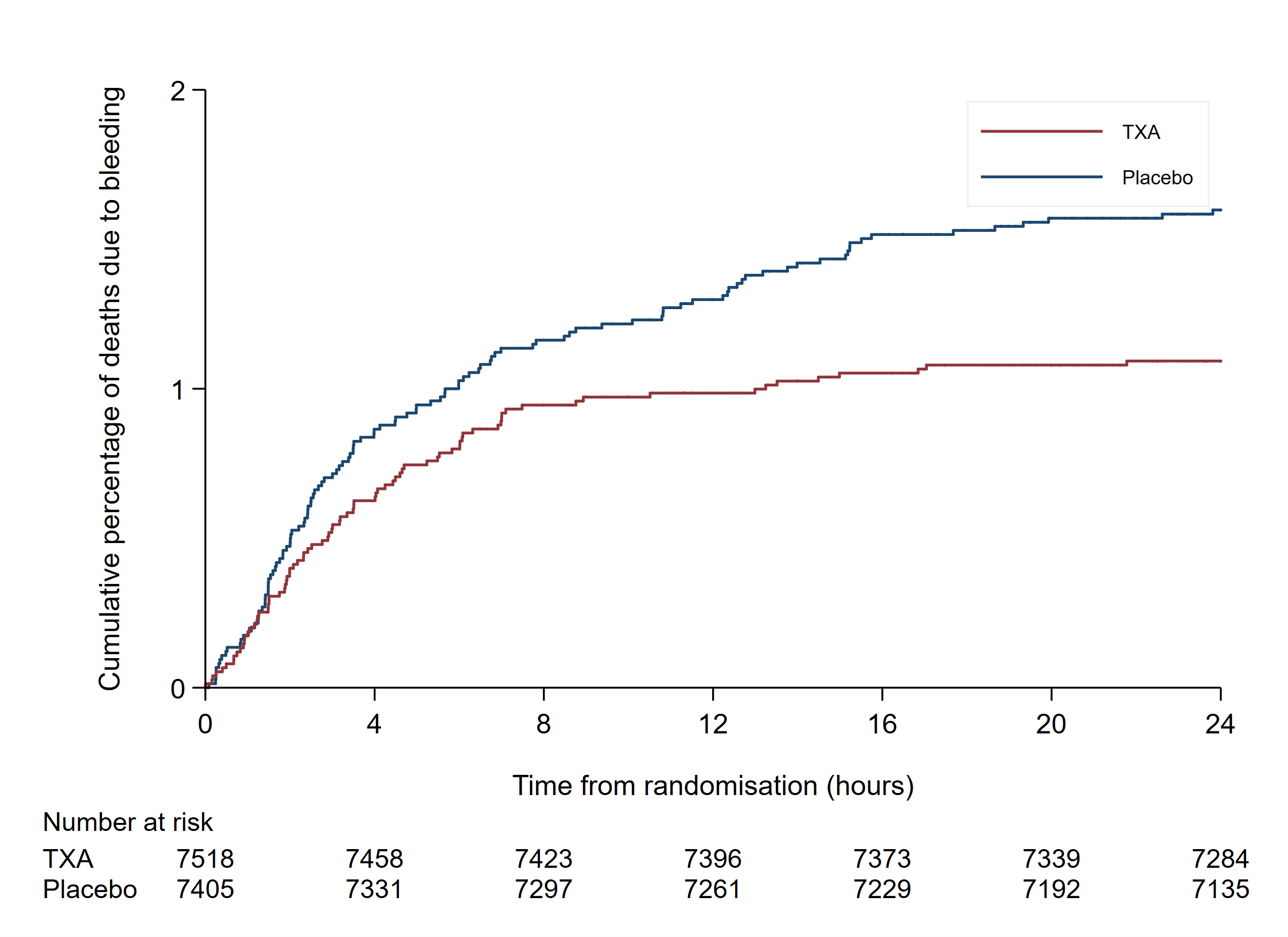
**

Table S3: **Other causes of death and all-cause mortality**

| Cause of death | TXA  (n = 6 000) | Placebo  (n = 6 000) | Risk ratio (95% CI) |
| --- | --- | --- | --- |
|  |  |  |  |
| Bleeding | NN (X%) | NN (X%) | X.XX (X.XX­–X.XX) |
|  |  |  |  |
| Thrombosis | NN (X%) | NN (X%) | X.XX (X.XX­–X.XX) |
|  |  |  |  |
| Organ failure | NN (X%) | NN (X%) | X.XX (X.XX­–X.XX) |
|  |  |  |  |
| Pneumonia | NN (X%) | NN (X%) | X.XX (X.XX­–X.XX) |
|  |  |  |  |
| Sepsis | NN (X%) | NN (X%) | X.XX (X.XX­–X.XX) |
|  |  |  |  |
| Malignancy | NN (X%) | NN (X%) | X.XX (X.XX­–X.XX) |
|  |  |  |  |
| Other | NN (X%) | NN (X%) | X.XX (X.XX­–X.XX) |
|  |  |  |  |
| All-cause | NN (X%) | NN (X%) | X.XX (X.XX­–X.XX) |
|  |  |  |  |
|  |  |  |  |

**Figure S3: Distribution of cause of death by days since randomisation**

| **Number of deaths** | | | | |  |  |  |  |  |  |  |  |  |  |  |  |  |  |  |  |
| --- | --- | --- | --- | --- | --- | --- | --- | --- | --- | --- | --- | --- | --- | --- | --- | --- | --- | --- | --- | --- |
|  |  |  |  |  |  |  |  |  |  |  |  |  |  |  |  |  |  |  |  |  |
|  |  |  |  |  |  |  |  |  |  |  |  |  |  |  |  |  |  |  |  |  |
|  |  |  |  |  |  |  |  |  |  |  |  |  |  |  |  |  |  |  |  |  |
|  |  |  |  |  |  |  |  |  |  |  |  |  |  |  |  |  |  |  |  |  |
|  |  |  |  |  |  |  |  |  |  |  |  |  |  |  |  |  |  |  |  |  |
|  |  |  |  |  |  |  |  |  |  |  |  |  |  |  |  |  |  |  |  |  |
|  |  |  |  |  |  |  |  |  |  |  |  |  |  |  |  |  |  |  |  |  |
|  |  |  |  |  |  |  |  |  |  |  |  |  |  |  |  |  |  |  |  |  |
|  |  |  |  |  |  |  |  |  |  |  |  |  |  |  |  |  |  |  |  |  |
|  |  |  |  |  |  |  |  |  |  |  |  |  |  |  |  |  |  |  |  |  |
|  |  |  |  |  |  |  |  |  |  |  |  |  |  |  |  |  |  |  |  |  |
| 0 | 1 | 2 | 3 | 4 | 5 | 6 | 7 | 8 | 9 | 10 | 11 | 12 | 13 | 14 | 15 | 16 | 17 | 18 | 19 | 20 |
|  |  |  |  |  |  |  |  |  |  | **Days** | |  |  |  |  |  |  |  |  |  |

Notes: Bars will be stacked to show number of deaths due to bleeding, thrombosis, organ failure, pneumonia, sepsis, malignancy and other causes.

Table S4: **Death due to bleeding by subgroups**

| **Subgroup** | **TXA** | | **Placebo** | | **Crude risk ratio (95% CI)** | **Adjusted risk ratio**‡ **(95% CI)** |
| --- | --- | --- | --- | --- | --- | --- |
|  | Death due to bleeding | Received TXA | Death due to bleeding | Received Placebo |
|  |  |  |  |  |  |  |
| **Time to treatment** | |  |  |  |  |  |
| ≤ 3h | NN (X%) | N NNN | NN (X%) | N NNN | X.XX (X.XX­–X.XX) | X.XX (X.XX­–X.XX) |
| >3h | NN (X%) | N NNN | NN (X%) | N NNN | X.XX (X.XX­–X.XX) | X.XX (X.XX­–X.XX) |
| *(p=0.XXX)*† |  |  |  |  |  |  |
| Continuous | NN (X%) | mean (SD) | NN (X%) | mean (SD) | X.XX (X.XX­–X.XX) | X.XX (X.XX­–X.XX) |
| *(p=0.XXX)*† |  |  |  |  |  |  |
|  |  |  |  |  |  |  |
| **Location of bleeding** | |  |  |  |  |  |
| Upper GI | NN (X%) | N NNN | NN (X%) | N NNN | X.XX (X.XX­–X.XX) | X.XX (X.XX­–X.XX) |
| Lower GI | NN (X%) | N NNN | NN (X%) | N NNN | X.XX (X.XX­–X.XX) | X.XX (X.XX­–X.XX) |
| *(p=0.XXX)*† |  |  |  |  |  |  |
|  |  |  |  |  |  |  |
| **Suspected variceal bleeding and comorbid liver disease** | | | | | | |
| Yes | NN (X%) | N NNN | NN (X%) | N NNN | X.XX (X.XX­–X.XX) | X.XX (X.XX­–X.XX) |
| No/unknown | NN (X%) | N NNN | NN (X%) | N NNN | X.XX (X.XX­–X.XX) | X.XX (X.XX­–X.XX) |
| *(p=0.XXX)*† |  |  |  |  |  |  |
|  |  |  |  |  |  |  |
| **Clinical Rockall score** | | |  |  |  |  |
| 1 - 2 | NN (X%) | N NNN | NN (X%) | N NNN | X.XX (X.XX­–X.XX) | X.XX (X.XX­–X.XX) |
| 3 - 4 | NN (X%) | N NNN | NN (X%) | N NNN | X.XX (X.XX­–X.XX) | X.XX (X.XX­–X.XX) |
| 5 - 7 | NN (X%) | N NNN | NN (X%) | N NNN | X.XX (X.XX­–X.XX) | X.XX (X.XX­–X.XX) |
| *(p=0.XXX)*† |  |  |  |  |  |  |
|  |  |  |  |  |  |  |

Notes: Time to treatment represents the time between symptom onset and randomisation.

† P-values from adjusted tests of interaction in a Poisson model with robust error variance to assess evidence for whether the effect of treatment differs across subgroup categories.

‡ Time to treatment analysis adjusted for age, systolic blood pressure, heart rate, location of bleeding, suspected active bleeding and suspected variceal bleeding.

Location of bleeding analysis adjusted for time to treatment, age, systolic blood pressure, heart rate and suspected variceal bleeding.

Cause of bleed analysis adjusted for time to treatment, age, systolic blood pressure, heart rate and suspected active bleeding.

Bleed severity analysis adjusted for time to treatment, suspected variceal bleeding, suspected active bleeding and anticoagulant therapy.

Table S5: **Need for surgical, endoscopic and radiological interventions and blood transfusion**

|  | **TXA**  (n = 6,000) | **Placebo**  (n = 6,000) | **Risk ratio (95% CI)** |
| --- | --- | --- | --- |
|  |  |  |  |
| **Endoscopic, radiological and surgical procedures** | | |  |
|  |  |  |  |
| Diagnostic endoscopy | NN (X%) | NN (X%) | X.XX (X.XX­–X.XX) |
|  |  |  |  |
| Therapeutic endoscopy | NN (X%) | NN (X%) | X.XX (X.XX­–X.XX) |
|  |  |  |  |
| Diagnostic radiological procedure | NN (X%) | NN (X%) | X.XX (X.XX­–X.XX) |
|  |  |  |  |
| Therapeutic radiological procedure | NN (X%) | NN (X%) | X.XX (X.XX­–X.XX) |
|  |  |  |  |
| Surgical intervention | NN (X%) | NN (X%) | X.XX (X.XX­–X.XX) |
|  |  |  |  |
| Any surgical, endoscopic or radiological intervention | NN (X%) | NN (X%) | X.XX (X.XX­–X.XX) |
|  |  |  |  |
| **Blood product transfusion** |  |  |  |
|  |  |  |  |
| Whole blood/red cells | NN (X%) | NN (X%) | X.XX (X.XX­–X.XX) |
|  |  |  |  |
| Frozen plasma | NN (X%) | NN (X%) | X.XX (X.XX­–X.XX) |
|  |  |  |  |
| Platelets | NN (X%) | NN (X%) | X.XX (X.XX­–X.XX) |
|  |  |  |  |
| Any transfusion | NN (X%) | NN (X%) | X.XX (X.XX­–X.XX) |
|  |  |  |  |
| **Blood product units transfused** |  |  |  |
|  |  |  |  |
| Whole blood/red cells | mean (SD) | mean (SD) | Diff in means (X.X­ – X.X) |
|  |  |  |  |
| Frozen plasma | mean (SD) | mean (SD) | Diff in means (X.X­ – X.X) |
|  |  |  |  |
| Platelets | mean (SD) | mean (SD) | Diff in means (X.X­ – X.X) |
|  |  |  |  |
| Any units transfused | mean (SD) | mean (SD) | Diff in means (X.X­ – X.X) |
|  |  |  |  |

‡ Katz index of Independence in Activities of Daily Living

Table S6: **Thromboembolic events, complications and self-care capacity**

|  | **TXA**  (n = 6 000) | **Placebo**  (n = 6 000) | **Risk ratio (95% CI)** |
| --- | --- | --- | --- |
|  |  |  |  |
| **Thromboembolic events** |  |  |  |
|  |  |  |  |
| Any event | N (X%) | N (X%) | X.XX (X.XX­–X.XX) |
|  |  |  |  |
| Venous events (DVT, PE) | N (X%) | N (X%) | X.XX (X.XX­–X.XX) |
|  |  |  |  |
| DVT | N (X%) | N (X%) | X.XX (X.XX­–X.XX) |
|  |  |  |  |
| PE | N (X%) | N (X%) | X.XX (X.XX­–X.XX) |
|  |  |  |  |
| Arterial events (MI, stroke) | N (X%) | N (X%) | X.XX (X.XX­–X.XX) |
|  |  |  |  |
| MI | N (X%) | N (X%) | X.XX (X.XX­–X.XX) |
|  |  |  |  |
| Stroke | N (X%) | N (X%) | X.XX (X.XX­–X.XX) |
|  |  |  |  |
| **Complications** |  |  |  |
|  |  |  |  |
| Renal failure | N (X%) | N (X%) | X.XX (X.XX­–X.XX) |
|  |  |  |  |
| Hepatic failure | N (X%) | N (X%) | X.XX (X.XX­–X.XX) |
|  |  |  |  |
| Respiratory failure | N (X%) | N (X%) | X.XX (X.XX­–X.XX) |
|  |  |  |  |
| Cardiac event | N (X%) | N (X%) | X.XX (X.XX­–X.XX) |
|  |  |  |  |
| Sepsis | N (X%) | N (X%) | X.XX (X.XX­–X.XX) |
|  |  |  |  |
| Pneumonia | N (X%) | N (X%) | X.XX (X.XX­–X.XX) |
|  |  |  |  |
| Seizure | N (X%) | N (X%) | X.XX (X.XX­–X.XX) |
|  |  |  |  |
| **Self-care capacity** |  |  |  |
|  |  |  |  |
| Katz ADL score‡ | mean (SD) | mean (SD) | Diff in means (X.X­ – X.X) |
|  |  |  |  |
|  |  |  |  |
| **Days in ICU or HDU** |  |  |  |
| Number of days | mean (SD) | mean (SD) | Diff in means (X.X­ – X.X) |
|  |  |  |  |

Notes:

DVT = deep vein thrombosis, HDU = High Dependency Unit; ICU = Intensive Care Unit;

PE = pulmonary embolism, MI = myocardial infarction

Table S7: **Adverse events**

| **Type of adverse event** | **TXA**  (n = 6 000) | **Placebo**  (n = 6 000) | **Risk ratio (95% CI)** |
| --- | --- | --- | --- |
|  |  |  |  |
| MedDRA system organ class groups † | N (X%) | N (X%) | X.XX (X.XX­–X.XX) |
|  |  |  |  |

Notes:

† AE’s, SAE, SUSAR grouped by MedDRA® system organ class codes
